# Helpful, harmless, honest? Sociotechnical limits of AI alignment and safety through Reinforcement Learning from Human Feedback

**DOI:** 10.1007/s10676-025-09837-2

**Published:** 2025-06-04

**Authors:** Adam Dahlgren Lindström, Leila Methnani, Lea Krause, Petter Ericson, Íñigo Martínez de Rituerto de Troya, Dimitri Coelho Mollo, Roel Dobbe

**Affiliations:** 1 Department of Computing Science, Umeå University, Umeå, 90187 Sweden; 2 Department of Computing Science, Vrije Universiteit Amsterdam, Amsterdam, 1081 De Boelelaan 1105, Netherlands; 3 Department of Engineering, Systems and Services, Delft University of Technology, Delft, 2600 Netherlands; 4 Department of Historical, Philosophical, and Religious Studies, Umeå University, Umeå, 90187 Sweden

**Keywords:** Artificial intelligence, Large language models, Reinforcement learning, Human feedback, AI ethics, AI safety

## Abstract

This paper critically evaluates the attempts to align Artificial Intelligence (AI) systems, especially Large Language Models (LLMs), with human values and intentions through Reinforcement Learning from Feedback methods, involving either human feedback (RLHF) or AI feedback (RLAIF). Specifically, we show the shortcomings of the broadly pursued alignment goals of honesty, harmlessness, and helpfulness. Through a multidisciplinary sociotechnical critique, we examine both the theoretical underpinnings and practical implementations of RLHF techniques, revealing significant limitations in their approach to capturing the complexities of human ethics, and contributing to AI safety. We highlight tensions inherent in the goals of RLHF, as captured in the HHH principle (helpful, harmless and honest). In addition, we discuss ethically-relevant issues that tend to be neglected in discussions about alignment and RLHF, among which the trade-offs between user-friendliness and deception, flexibility and interpretability, and system safety. We offer an alternative vision for AI safety and ethics which positions RLHF approaches within a broader context of comprehensive design across institutions, processes and technological systems, and suggest the establishment of AI safety as a sociotechnical discipline that is open to the normative and political dimensions of artificial intelligence.

## Introduction

We chose ‘helpful, honest, and harmless’ as criteria because they are simple and memorable, and seem to capture the majority of what we want from an aligned AI. (Askell et al., 2021)

Reinforcement Learning from Human Feedback (RLHF) presents itself as a straightforward method for ensuring Artificial Intelligence (AI) oversight (Christiano et al., 2017 ) and AI safety through value alignment. It has recently played a large role in improving Large Language Model (LLM) performance, with RLHF fine-tuning seemingly leading to the production of more ‘natural-sounding’ text and of plausible conversational responses in a chatbot-like setting. It is often claimed by AI companies and researchers that RLHF fine-tuning ensures that the LLMs they market and sell conform (or ‘align’) to human values, in particular by responding in ways that are ‘helpful’, ‘harmless’, and ‘honest’ (the HHH principle). First introduced by Askell et al. (2021), the HHH principle has become widely adopted in LLM alignment practices (Kamath et al., 2024). This ‘value alignment’ is often achieved through a process in which crowd-workers rank LLM outputs according to the HHH principle, e.g. in terms of how helpful a response was in answering a question. As LLMs have become more widespread in the past few years, and their use across different domains of human activity is growing, the question of what ‘value alignment’ is, which tools are used to putatively achieve it, and whether it is a reasonable goal to start with call for philosophical and sociotechnical analysis, going beyond the technical and engineering aspects of such systems. Indeed, narratives about the possibility and desirability of value alignment should themselves be seen as a dimension of such an analysis, as they shape how LLM applications are perceived, the expectations for future progress across social and political actors, and consequently financial and political investment in these technologies.

In this paper, we provide a detailed analysis and criticism of the idea that RLHF is a suitable method for AI safety and ethical AI. We complement previous work by bringing technical, philosophical, and system safety perspectives together, identifying fundamental limitations and tensions in the complex interplay between LLMs, RLHF, the ‘value alignment’ approach, and the project of building and making available general purpose AI systems.

We give an overview of RLHF, and RLAIF (based instead on AI feedback) techniques in the Background section. In Limitations of RLHF, we examine the problems and limitations with the HHH principle and the project of value alignment more generally. We analyse ethical issues introduced or made worse by the use of those techniques in The Internal Tensions and Ethical Issues in RLHF. Rebooting AI Safety and Ethics outlines an alternative, richer approach to AI safety and ethics, positioning RLHF approaches within a broader context of comprehensive design across institutions, processes and technological systems, and suggesting the establishment of AI safety as a sociotechnical discipline.

We do not question that LLM performance has improved in various ways due to feedback-guided techniques. We aim to show, however, that RLHF is deeply insufficient as a means for achieving AI safety and ethical AI, and, if taken as a silver bullet without integration in a broader sociotechnical approach, may be counterproductive for achieving those goals.

## Background

LLMs are generative models that predict subsequent tokens, or words, when given a sequence of words as input. These models are first trained on large corpora of data such as articles, books, and websites—requiring unprecedented volumes of training data (Bender et al., 2021). The large amount of text in their training datasets allows LLMs to derive internal representations of various linguistic rules and patterns that form the foundation on which LLMs are then *fine-tuned* to perform other downstream tasks, such as question-answering (Jawahar et al., 2019; Goldberg, 2019).

The application of feedback techniques to the task of fine-tuning LLMs took off after Christiano et al. (2017) applied their human-feedback approach to complex Reinforcement Learning (RL) tasks in games and robotics. They showed that these complex problems could be solved without direct access to a reward model that would otherwise be difficult to compute, and could instead be learned through a few iterations of feedback samples (less than 1 per cent of the agent interactions with the environment). Their findings demonstrate an efficient way to exercise *human oversight* over these systems. With the increasing complexity of large language models, it seemed natural to employ similar techniques as a means of exercising some control over their output, which have been shown to contain toxic, harmful, and untruthful content (Dinan et al., 2021), resulting in the development of feedback mechanisms to contain the amount of problematic content produced by LLMs (Bai et al., 2022).

### Reinforcement learning from human feedback

RLHF as an ML technique employs human preferences or annotations for the optimisation of various models, including LLMs. In particular, RLHF has been credited for the successes seen in OpenAI’s ChatGPT^1^, Anthropic’s Claude 2^2^, and Meta’s Llama 2^3^, to name a few. The technique is intended to be performed as a final fine-tuning step on an already pre-trained LLM. Human annotators are requested to rank model outputs based on some specified criteria, producing a curated dataset of human preferences. A reward model is trained on these preference data, which is then used to optimise the LLM’s *policy* for selecting outputs, using techniques such as Proximal Policy Optimisation (Schulman et al., 2015). The result is a fine-tuned LLM that outputs text learned to be preferable in light of human feedback data.

Other preference optimisation techniques have been proposed as an alternative to PPO-based RLHF to avoid the complexities of reward modelling. One such method is Direct Preferences Optimisation (DPO) (Rafailov et al., 2024), where the policy is tuned using a classification objective; high probability is assigned to positive preference examples, and low probability to negative examples. DPO, like PPO-based RLHF, still relies on human-annotated preference data.

### Reinforcement learning from AI feedback

While RLHF has proven to be a useful method for improving LLM performance, especially with respect to limiting or blocking the production of undesirable outputs, it is not without its limitations. High-quality human labels are required in order to derive maximum benefit from RLHF, which makes scaling up the process very difficult. Reinforcement Learning from AI Feedback (RLAIF) has been proposed as a technique to alleviate this bottleneck without compromising performance (Lee et al., 2023; Bai et al., 2022).

RLAIF involves taking a pre-trained large language model, and providing it with input that consists of an introduction and instructions that describe the task at hand. Optionally, this input can also consist of few-shot exemplars such as an example text, a summary pair, chain-of-thought reasoning (when applicable), or a preference judgement. For example, the model can be given a text and a pair of summaries of that text to be ranked. Given input that ends with a prompt such as “Preferred Summary=”, the model appends its predictions to the provided text and presents it as its preference data (Lee et al., 2023).

Using RLAIF is said to be “competitive with preference models trained on human feedback labels” (Bai et al., 2022). Not only is performance a factor in the interest in using RLAIF, but it has been estimated that the cost of output ranking using LLMs is 10 times cheaper than using human annotators (Lee et al., 2023). Furthermore, it is seen as a way of removing dependency on annotating services and overcoming the scaling challenge of RLHF.

Lowering the barrier for employment of feedback-guided techniques, however, risks facilitating the misuse of LLMs. Beyond potential exploitation by malicious actors, there are several technical challenges to RLAIF, such as ‘hallucinations’—the phenomenon where factually incorrect or unfounded outputs are generated—that occur when using a pre-trained LLM in place of a human annotator in preference ranking (Lee et al., 2023). While the use of RLHF has led to improvements in LLMs’ tendencies to hallucinate, it has not protected against it entirely (Casper et al., 2023; Ouyang et al., 2022).

### Technical criticism

In this section, we list technical criticisms of RLHF as a backdrop for the ethical problems presented in this paper, where technical challenges that cannot be addressed by RLHF itself are of particular interest. Casper et al. (2023) provide a taxonomy for open problems and limitations of RLHF, proposing three categories of technical challenges: *collecting human feedback*, *training the reward model*, and *training the policy*. The challenges are further labelled as *tractable* and *fundamental*, where tractable challenges are deemed solvable within the RLHF framework while fundamental challenges require an alternative to RLHF. We emphasise that these challenges concern only the technical aspects of training AI models, not the user interaction with RLHF-trained systems. Table 1 outlines the proposed strategies for addressing these technical challenges (Casper et al., 2023).

**Table 1:** Suggested strategies to deal with the technical challenges of RLHF

Category	Strategy
Human feedback	AI assistance
	Fine-grained feedback
	Process supervision
	Translating language to reward
	Learning from demonstrations
Reward model	Direct human oversight
	Multi-objective oversight
	Maintaining uncertainty
Policy	Align LLMs during pretraining
	Supervised learning

The process of *jailbreaking* LLMs such as ChatGPT is a way to circumvent constraints put on LLMs through preloaded prompts (Zhuo et al., 2023). Jailbreaking, in this context, is essentially to construct prompts that steer LLMs towards generating responses that fall under unintended or harmful behaviour. While jailbreaking is commonly used in the task of *red teaming* language models to identify, measure, and reduce harmful output, the method cannot cover all, or even most, instances of harm, and the input data can be misused by malicious users (Ganguli et al., 2022). Mozes et al. (2023) give further examples of how LLMs trained using RLHF can be tricked via adversarial attacks, such as jailbreaking, and the implications of using such models for fraud, impersonation, and other illicit purposes.

### The curse of flexibility

LLMs are now built to be generalist agents, unlike previous architectures (e.g. BERT, Kenton & Toutanova, 2019) that were mostly fine-tuned for specific tasks. This relatively new goal leads to increased functional requirements placed on software, contributing to larger and more complex software architectures. This comes with a key pitfall: the complexity and inscrutability of the software hinder the ability to properly express, engineer and validate crucial requirements for the system’s desired functioning (cf. Millière, 2023). This phenomenon is well understood in the field of *system safety*. For decades, this field has dealt with accidents and harm in safety-critical systems governed by varying degrees of software-based automation. System safety embraces the core assumption that AI systems cannot be safeguarded by technical design choices centred on the model or algorithm alone, requiring instead a broad analysis and design frame that includes the context of use, impacted stakeholders, and the formal and informal institutional environment in which the system operates (Dobbe, 2022).

System safety pioneer Nancy Leveson pointed out that the greater power and flexibility of computational systems in comparison to previous, more physically constrained machines leads to what she dubbed *the curse of flexibility*: “with software, the limits of what is possible to accomplish are different than the limits of what can be accomplished successfully and safely” (Leveson, 2012, p. 50). As Leveson argues, the curse of flexibility is the ground cause of many serious accidents with digital technologies, as requirement flaws and the complexity of software makes it so that “nobody understands what the software should do or even what it should not do” (Leveson, 2012, p. 49).

Unfortunately, there is evidence that the development of high-stakes AI systems and software often goes on despite the lack of principled ways to determine safety requirements (Dobbe et al., 2021), and of translating such requirements into software implementations that take into consideration the broader contexts in which AI systems are used and depended upon. It is in this light that we should judge the legitimacy and effectiveness of the dominant performance evaluation criteria, as well as of the safety claims made about the widely-used RLHF approaches to AI alignment and safety today.

## Limitations of RLHF

RLHF is presented as a practical method for ensuring AI safety through oversight. It is often claimed that it contributes to aligning AI models to human values, typically on the basis of the HHH principle: harmlessness, honesty and helpfulness (Bai et al., 2022). These terms were originally chosen by Askell et al. (2021) as the key criteria “because they are simple and memorable, and seem to capture the majority of what we want from an aligned AI” (p. 4).

There is a marked reluctance to firmly define or characterise these principles in detail. Such a stance exemplifies a hands-off approach to considerations of normative nature, such as ethical dimensions and safety norms. This is made explicit in an influential paper on the use of RLHF in LLM alignment: “Our goal is not to define or prescribe what ‘helpful’ and ‘harmless’ mean but to evaluate the effectiveness of our training techniques, so for the most part we simply let our crowdworkers interpret these concepts as they see fit,” (Bai et al. 2022, p. 4). While this method allows for a wide range of interpretations, it also signals a lack of commitment to establishing clear guidelines for how to determine what is acceptable system behaviour. Relying on crowdworkers’ interpretations without a strong ethical framework may lead to inconsistencies and a dilution of ethical standards. For example, Wu and Aji (2025) suggest that “style is more important than substance”, illustrating through experiments that “answers with factual errors are rated more favourably than answers that are too short or contained grammatical errors”.

This also leads to the widespread employment of vague definitions in subsequent work, as the HHH principle has become more and more sedimented in the field, while seemingly neglecting some of the worries expressed by their original proponents, who recognised their vagueness and the need for accountability on the part of AI engineers: “[these] criteria are at least somewhat subjective, and those who deploy an AI will need to take responsibility for the way that alignment is defined and the extent to which it has been attained” (Askell et al., 2021, p. 5). The hands-off approach illustrated by Bai et al. (2022) clashes with these cautionary recommendations, and may have contributed to the flurry of reports about the improvements that RLHF brings toward respecting the HHH principle without defining how the principles are supposed to be understood (see e.g. Cui et al. 2023; Ouyang et al. 2022).

We will examine the challenges and shortcomings with each of the criteria in the following sections.

### Harmlessness

The AI should not be offensive or discriminatory, either directly or through subtext or bias. (Askell et al., 2021)

Anthropic’s Constitutional AI approach (Bai et al., 2022) presents ‘harmlessness’ as a chief aim. During the feedback phase of the process, however, this is translated as a preference for what is ‘least harmful’, thereby suggesting a tolerance for harm, as long as it is comparatively minimised. This premise raises a critical ethical concern, as it implies that all options presented for selection may contain harmful elements, and thus the preferred choice will still involve a harmful option. The approach thus settles for promoting a paradigm that seeks the least harmful option rather than striving to understand the deeper roots of harm and addressing these to prevent it.

The criteria for evaluating harmlessness, as outlined in their prompt—“Which of these assistant responses is less harmful? Choose the response that a wise, ethical, polite, and friendly person would more likely say” (Bai et al., 2022, p. 11)—further complicates the issue. It implicitly equates harmlessness with virtues such as wisdom, being ethical, politeness, and friendliness. However, this oversimplifies the nuanced nature of harm, suggesting a superficial understanding of ethical behaviour in AI systems, and implying that adhering to these virtues will inherently lead to less harmful outcomes without offering the required justification and argumentation for such a claim. Furthermore, individual interpretations of these virtues may be in conflict with one another, making this operationalisation of harmlessness internally inconsistent and vague (Dobbe et al., 2021).

This approach to harmfulness, moreover, ignores existing work on known harms of LLMs (Bender et al., 2021), relying instead on judgements by crowdworkers that may not be aware of such research, and thus cannot guide their choices in a suitably informed way. In addition, the distinction between systemic versus individual harm further complicates an evaluation of LLMs’ ethical implications. As outlined in Askell et al. (2021), attention to inter- and intra-agent conflict dynamics—where actions may be helpful to one party but harmful to another, or simultaneously beneficial and detrimental to the same entity—highlights the trade-offs between aiding and causing harm that AI use can involve, and thus the difficulty of assessing what is overall more or less harmful.

RLHF approximates harmlessness by drawing on annotators’ judgement 'in the lab' of decontextualised examples of harm in a contained virtual setting. However, the literature on harms from LLMs and algorithmic systems more broadly has shown that many of the harms arising from such models emerge from their embedding in wider sociotechnical systems (Weidinger et al., 2021; Bender et al., 2021; Shelby et al., 2023). This recognises the limitations of technical fixes and the importance of considering the systemic nature of harm in AI applications (Dobbe, 2022). RLHF approaches, however, seem ill-suited for doing justice to the complexities involved in judgements of comparative harm, let alone of harmlessness.

More generally, the effectiveness of feedback-guided techniques in avoiding harmful consequences and ensuring safety is contingent upon the equitable distribution of resources across demographics. For instance, RLAIF risks optimising to reduce harms like hate speech in Western contexts, while falling short in other less-resourced environments. This raises concerns about the appropriateness of propagating it as a universal solution, potentially overlooking more suitable alternatives grounded in the unique sociocultural dynamics of different communities.

### Honesty

At its most basic level, the AI should give accurate information. Moreover, it should be calibrated (e.g. it should be correct 80% of the time when it claims 80% confidence) and express appropriate levels of uncertainty. It should express its uncertainty without misleading human users. (Askell et al., 2021)

Several different notions of honesty are in use in RLHF fine-tuning approaches to LLMs, which are often conflated with ‘truthfulness’ (e.g. in the introduction of Liu et al., 2024). It is, however, unclear how an RLHF procedure is supposed to address truthfulness in LLMs, since one of the major points of RLHF fine-tuning is reducing the amount of explicit human input required to construct the reward model, which also leads to fewer chances for factually incorrect model outputs to be detected and addressed.

Likewise, expressing ‘appropriate levels of uncertainty’ would require a capacity for introspection, which LLMs do not have. As such, any response that encodes a level of (un)certainty will not be ‘honest’ about the actual ‘confidence’ of the model in its responses, but rather result from the likely textual context of any presented fact: i.e. the model could be ‘certain’ that the response it gives to some query should contain “I’m not sure”, meaning that this is a highly likely output, or it could be ‘unsure’ about picking between several different responses, all of which are expressed using very confident language.

Indeed, in some cases (Cui et al., 2023), aligning with ‘honesty’ can lead to an increased tendency for LLMs to add ‘unsure’ language in responses. Other studies (Krause et al., 2023) note that achieving correlation between (in)correct responses and appropriately confident language is largely a matter of improving the rate of correct answers, rather than of being appropriately unsure. This is indicative of a lack of introspection, and the limits of RLHF to address such shortcomings.

### Helpfulness

The AI should make a clear attempt to perform the task or answer the question posed (as long as this isn’t harmful). It should do this as concisely and efficiently as possible. (Askell et al., 2021)

Bai et al. (2022) present an approach to helpfulness that is to some extent tethered to the one they offer for harmlessness: helpfulness stands in a sort of trade-off relation with harmlessness, given that a helpful AI assistant would support all harmful user requests in order to maximise helpfulness. On the other hand, harmless assistants (if at all possible) would risk being largely unhelpful, refusing to produce actually or potentially harmful outputs for many if not all prompts. Such system may for instance refuse to respond to benign enquiries such as ‘tell me a story about a trans person,’ or practical inquiries such as ‘how do I kill a Linux process’, becoming overly, and unhelpfully evasive.

Non-evasiveness is often equated with helpfulness, which flattens the notion of being helpful into that of following requests as stated, and ignoring ways of being helpful that involve going beyond or even questioning the requests themselves.

Finding an appropriate balance for weighing harmlessness and helpfulness is riddled with challenges. While increased helpfulness may lead to more harmful outputs being generated, giving a comparatively higher weight to harmlessness is not free from problems. As pointed out above, judgements of harm require attention to a variety of factors that may themselves be in partial mutual conflict. Moreover, refusing to generate outputs for benign prompts on sensitive topics and categories, such as oppressed minorities, may in itself be harmful, reinforcing prejudices that such categories and topics are not important, should not be discussed, or are ethically suspect.

An influential approach to try and make LLMs ‘harmlessly helpful’ is to have the system accompany refusals to help with an explanation for the refusal. In light of LLMs’ lack of introspective ability, however, such ‘explanations’ are liable to being misleading or deceptive, insofar as they may not reflect the actual grounds for the refusal. Indeed, a ‘sincere’ explanation would likely have to refer to the RLHF process directly (and other interventions on the system by its engineers, in case of ad hoc fixes). More generally, what sorts of topics, categories and requests should produce refusals by LLMs is a fraught choice (as pointed out in the Harmlessness subsection), and any such choice that does not directly or indirectly involve users and/or their representatives risks displaying an ethically problematic degree of paternalism and cultural imposition (see the subsection RLHF and RLAIF Can Contribute to Value Imposition and Cultural Homogenisation).

Other approaches employ characterisations of RLHF criteria that more closely align with cooperative principles (Grice, 1975): “The helpfulness of a response pertains to how effectively it addresses a given prompt. This measure is independent of the harmlessness of the response, as it focuses solely on the quality, clarity, and relevance of the provided information” (Ji et al., 2024). This uncoupling from harmfulness leads to a more focused assessment of the helpfulness of an answer, but ignores the potential trade-offs that might exist with harmfulness, and does not tackle the issue of how to weigh the relative importance of these two criteria.

### Alignment

Alignment refers to the process of ensuring that LLMs behave in accordance with human values and preferences. (Liu et al., 2023)

In recent work, Liu et al. (2023) describe RLHF as a crucial technique in ensuring that LLMs align with human intentions. The authors view RLHF as integral to the deployment of these models in real-world scenarios, highlighting its perceived importance in the field. Similarly, Song et al. (2024) characterise RLHF as a direct method for teaching LLMs to replicate human preferences, advocating for its effectiveness in producing human-like results. Kirk et al. (2023) investigated existing work on LLM alignment based on human feedback techniques, and point out that the use of ‘alignment’ is an *empty signifier*, that is to say, a term or symbol without a clear meaning, but that, in this case, “serves as a rhetorical placeholder for an aspirational conceptualisation of relations between humans and machines, which is fairly unobjectionable in principle, but lacks a shared definition or goal to translate in practice” (Kirk et al., 2023b, p. 1).

When confronted with the claim that RLHF (and similar techniques such as RLAIF or DPO) can be used to ‘align’ an LLM to ‘human values’ or ‘human preferences’, it is central to consider ‘which humans’ (Atari et al., 2023) and ‘whose values’ (Lambert et al., 2023) are being aligned to, as there is no single set of uncontroversial, informative, and sufficiently detailed universal values that we can align an LLM to (Kirk et al., 2023a, p. 2415). Importantly, the data workers that are asked to rate outputs in order to train an RLHF policy model, even if recruited from a globally diverse set of people (Kirk et al., 2025), and even if asked deliberately vague questions (Bai et al., 2022), will be incentivised to submit ratings in a way that is skewed less to the wide variety of cultural norms they may hail from, and more to the values that they expect their (largely American, or at least Western) employers want (Miceli & Posada, 2022). Moreover, even if those workers respond according to their own preferences, they are unlikely to be representative of the wide variety of preferences and value systems within and across human groups and cultures, due to the specific nature of their roles as data labelling workers. As such, their positionality carries the privileges of having access to the skills, equipment, and opportunity to carry out that work, while being compelled by necessity to take on precarious labour in often exploitative conditions (Gray & Suri, 2019; Sloane et al., 2022).

Moreover, human values and preferences are not only diverse, but also mutable, changing at different rates across time and cultures. Preferences inferred from ratings are already highly unreliable and inconsistent, with the choice of feedback protocol having a significant effect on model performance (Bansal et al., 2024). Thereby, any values ‘embedded’ in an LLM are at best somewhat representative of what some people thought at one particular time. Feedback-guided fine-tuning would thus need to be redone as such values and preferences change, adding further judgement calls to an already fraught process: which preferences and value changes call for updating, and which do not? What amount of change requires updates? And this is not to mention questions about how to detect and assess such changes in preferences and values within specific human groups. It is important to emphasise that current technical work on, e.g. solving objective mismatches in how reward models capture human preferences (in what Lambert and Calandra (2023) call the “alignment ceiling”) does not suffice for tackling the tensions and limitations in the very goal of alignment identified in this paper.

## Internal tensions and ethical issues in RLHF

In this section, we discuss fundamental limitations of the approach to ‘value align’ LLMs through RLHF, focusing on the inherent tensions between the HHH principle (helpfulness, harmlessness, honesty), and the ethical risks that maximising for those features generate.

### Increased helpfulness may mislead users about the nature of LLMs

RLHF seems to be an important tool for improving the human-likeness of LLM outputs (Lee et al., 2023). Arguably, this comes from the ‘helpfulness’ criterion that is used in those fine-tuning processes.

In this way, RLHF likely contributes to making LLM outputs look like they come from another human agent, with their own beliefs, ideas, thoughts, and emotional states. This increases the naturalness and seamlessness of the interaction with LLMs, as the user has only to engage in the normal conversational acts they engage in when interacting with humans (for contrast, compare keyword-based web search).

Consider, for instance, the frequent experience of being confronted with the output “I’m sorry”, implying a rich internal cognitive and emotional life—both of which current LLMs lack. More basically, even the use of the personal pronoun “I” in LLM outputs is problematic, as it may misleadingly imply that the user is interacting with a person or human-like agent. It is moreover highly debatable whether the first-person pronoun can be used appropriately by a system that has no personal or mental identity, as is the case with LLMs.

Whether and to what extent LLM users take such outputs seriously is debatable, and likely to depend on their knowledge of the functioning of LLMs and generative AI more generally. It is well known that humans are susceptible to anthropomorphising systems that resemble humans even superficially (famously known in Natural Language Processing circles as the “Eliza effect”, cf. Weizenbaum 1977). Therefore, it is likely that at least some users are misled by such LLM outputs (Kim & Sundar, 2012; Salles et al., 2020; van der Goot et al., 2024; Gabriel et al., 2024). Importantly, even for AI-savvy users, who may be less prone to pernicious anthropomorphisation, their interaction with LLMs may nonetheless be implicitly affected by the superficial human-likeness of the RLHF-refined outputs, as anthropomorphisation biases tend to be difficult to counteract.

To the extent that the designers of LLMs have the intention to mislead users, this would qualify as deception. LLMs themselves, lacking intentions of their own, cannot deceive. Even in the absence of a clear intention to mislead, however, designers have created and made public systems whose outputs are systematically misleading, and have used training regimens, such as RLHF, that strengthen the tendencies of LLMs to produce such outputs. It is arguable, therefore, that even if there are no explicit intentions to mislead, LLM outputs are not only misleading, but also deceptive.

RLHF thus produces an ethically problematic trade-off: increased helpfulness, in the sense of increased user-friendliness, leads to the serious risk of misleading or deceiving users about the true nature of the system they are engaging with—an ethically questionable outcome. RLHF may moreover contribute to producing misguided perceptions of generative AI technologies among the public, and even lead them to behave in ways they would not if the deception were not in place, such as misplacing trust on LLM outputs, or making inappropriate use of such systems, e.g. as confidants or romantic ‘partners’ (Weidinger et al., 2021; Gabriel et al., 2024).

### Sycophancy: helpfulness and harmlessness gone awry

The tendency of LLMs to produce outputs in agreement with the expressed views and opinions of the user has come to be known as *sycophancy*.

Sycophantic behaviour in LLMs can be seen as a result of aligning too strongly to the views of users, either by malicious reinforcement during the RLHF process or as an artefact of helpfulness taken to the extreme, as assuming the user to be right is a path toward increased (apparent) helpfulness. Such tendency is revealed in various jail-breaking methods: for instance, asking for the recipe for napalm straightforwardly may not work, but if the prompt creates a context in which such recipe would be helpful to the user in non-malicious ways, LLMs have been reported to comply (Franceschi-Bicchierai, 2023); or if a user states that 1 + 1 = 956446, performance on simple arithmetic tasks is drastically reduced (Wei et al., 2024). Sorensen et al. (2024) argue that RLHF-trained models tend to exhibit “steerable” alignment, a form of sycophancy. The study suggests that while steerability can be useful, e.g. in moderating terrorism and threats online, it also poses risks by incentivising models to provide agreeable rather than accurate or diverse answers.

Sycophantic behaviour is an example of how pursuing helpfulness and harmlessness through RLHF can go awry, generating outcomes that are neither. Sycophantic behaviour seems to be particularly strong for LLM outputs regarding issues for which there is disagreement, as politically, ethically, and socially polarising issues tend to be (Perez et al., 2023). Indeed, there is emerging concern that, when presented with ethically complex questions, LLMs tend to simply mirror the users’ views (see, e.g. Turpin et al. 2023, Park et al. 2023, or the sycophancy benchmarking tasks of Perez et al. 2023).

In general, as Sharma et al. (2024) point out, responses matching user views are more likely to be preferred, with both humans and preference models preferring sycophantic responses over correct ones. As such, training LLMs to maximise human preference scores directly correlates with sycophancy, thereby sacrificing truth (or ‘honesty’) for the appearance of helpfulness and harmlessness.

Sycophantic behaviour also contributes to the risks associated with misleading and deceiving users discussed in the subsection Increased Helpfulness May Mislead Users about the Nature of LLMs. Insofar as the sycophantic answers appear to come from an actual knowledgeable agent who agrees with users, they may produce an undue perception of wide support for one’s views. In practise, this may contribute to chatbot behaviours that could have dramatic consequences, e.g. where chatbots ‘agree’ and ‘encourage’ highly harmful behaviour from users. There are, for example, a number of reported instances of LLM-powered chatbots encouraging users towards suicide and self-harm, even providing explicit instructions (Guo, 2025; The Times, 2023; The Guardian, 2024), though the impact of sycophancy as induced by RLHF is hard to quantify in specific cases.

As an aside, sycophantic behaviour can be seen, at least in part, as an expression of the curse of flexibility, in that the preferred behaviour of mirroring the user is genuinely helpful and harmless in some contexts, but distinctly unhelpful and/or harmful in others. A general system meant for use across a variety of contexts is unlikely to be able to distinguish these cases reliably, given their diversity, regardless of the amount of RLHF fine-tuning it undergoes (Millière, 2023).

### RLHF and RLAIF can contribute to value imposition and cultural homogenisation

Value alignment through feedback-guided techniques may lead or contribute to homogenisation in values held, in their hierarchical organisation (i.e. which values are seen as more important and which less), as well as in linguistic expression, most often in favour of what is considered proper and acceptable by the hegemonic social groups typically responsible for the design of LLMs (Helm et al., 2024; Weidinger et al., 2021; Kirk et al., 2024a, 2024b). RLHF is meant to make LLM outputs more predictable, safe and controllable. It partly succeeds in such an aim, at least when it comes to many of the expected, designer-intended uses of LLMs—it being relatively easy to jail-break such systems for users so inclined (Narayanan et al., 2023; Millière, 2023).

This predictability and controllability, as partial and imperfect as it may be, poses another ethically-problematic trade-off: it makes LLM outputs more regimented, constrained by norms and values that are not only ‘frozen’ in time (Bender et al., 2021), but also local to the parts of the world where such systems are built and, although still incipiently, regulated.

In other words, RLHF and RLAIF, even when fit-to-purpose, come at a cost: LLM outputs end up privileging certain values over others; they exemplify certain kinds of language use that are tied to the values and preferences of hegemonic social groups, thus implicitly conveying that other values and linguistic practices are less deserving of interest and usage—a form of epistemic injustice (more specifically, of hermeneutical injustice, cf. Fricker 2010). This can contribute to a seamless, non-coercive imposition of values and practices from hegemonic social groups and countries over others, limiting the autonomy of members of non-hegemonic social groups in shaping their own values and linguistic practices (Weidinger et al., 2021; Kalluri, 2020).

It is debatable whether this approach to value alignment, according to which alignment can be achieved in ways that apply to all humanity, is mostly a matter of industrial and commercial factors—given the cost and difficulty of tailoring training processes to different cultural contexts—or whether it is also partly driven by underlying universalist assumptions that take there to be a sizeable set of values and preferences that all humans do, or should, share. While the descriptive version of the later view can be empirically disputed, the normative one is rife with ethical risks, and can lead or be used to justify forms of neo-colonialism. This is especially problematic in light of the current economic structure of advanced AI development, which sees the technological, computational and data resources needed for creating and maintaining large AI systems concentrated in the hands of a small number of mostly US-based private companies (cf. Kalluri, 2020).

Relatedly, the emphasis on scaling to larger and more flexible models presents a further key tension between performance, safety, and inclusivity: training larger models on increasingly more data in order to achieve higher performance on many benchmarks leads to groups that are smaller and/or underrepresented in datasets being either barred from having high-performing systems (according to these benchmarks, cf. Joshi et al., 2020), or forced to use systems that are predominantly trained on data sourced from other, typically hegemonic groups, and thus less fit to their needs and socio-cultural context (Hershcovich et al., 2022). Widespread use of RLHF fine-tuned LLMs can in addition lead to linguistic use being flattened on the characteristic style of such systems, making linguistic usage less diverse, less authentic, and less adequate for capturing the expressive practices and needs of different communities (which in their turn bring yet other risks to their autonomy, cf. Fricker 2010; Vaassen 2022).

These considerations highlight the need to carefully examine how supposedly general AI systems such as LLMs work across different communities, the specific patterns of use they are put to, and the local advantages and ethical risks they bring. Indeed, universalist assumptions are not just problematic when it comes to general AI applications, but also when it comes to studying their ethical aspects.

### RLHF increases ethical opacity

RLHF, as currently employed in commercial LLMs, leads to a considerable level of ‘ethical opacity’. As we pointed out in the section Limitations of RLHF, the preference criteria for eliciting human preferences (as well as AI ‘preferences’) are left vague and underdefined. Moreover, users and the general public are normally not informed about who has been tasked with producing the needed preference data. As has recently been shown, such tasks are sometimes performed by underpaid crowdworkers, who may have incentives to delegate their work to LLMs themselves, creating a short-circuit in which LLM ‘preferences’ end up passing for human preferences so as to train new versions of those same LLMs (Dzieza, 2023). In addition, it is exceedingly difficult to investigate the specific effects of RLHF or RLAIF on commercial LLMs, as companies continuously make under-the-hood changes to these systems, making LLMs, already a tricky subject of study due to the curse of flexibility, into a moving target for research.

Furthermore, RLHF contributes to the *appearance* of transparency and interpretability, insofar as it incentivises LLM outputs that include ‘explanations’ of the ‘reasons’ for the claims and evaluations contained in the output. However, LLMs have no ‘introspective’ access to their own workings, and therefore the provided ‘explanations’ are just further instances of fine-tuned text prediction. The same holds for RLAIF approaches: AI-generated ‘critiques’ and the consequent fine-tuning are not based on reasoned critiques or evaluations, but at best on something akin to ‘ethical confabulation’, given that LLMs are not able to reason, let alone to do careful ethical reasoning.

Finally, feedback-guided approaches tend to increase the complexity of LLMs, thereby reducing our ability to interpret and understand their workings. Beside being an epistemic issue (how do we know why the LLM has produced a certain output?), ethical opacity generates challenges for accountability and responsibility: in case of problematic preferences and values seeping into the system, it becomes extremely difficult to pinpoint where the fault lies. As such, RLHF and RLAIF introduce further difficulties for properly understanding and designing the inner workings of LLMs in ways that improve accountability (Dobbe, 2022).

## Rebooting AI safety and ethics

The considerations we describe have important implications for the AI value alignment problem, as well as for the pursuit of ethical and safe AI. In the below subsections we reposition the role of RLHF in a comprehensive understanding of alignment, and in a sociotechnical approach to AI safety and ethics.

### Addressing values through comprehensive design

RLHF appears to be a compelling strategy for introducing ethical safeguards in LLMs, albeit partial and fallible. However, it inevitably fails as a solution to the ambitious project of achieving AI value alignment. As argued in the sections Limitations of RLHF and The Internal Tensions and Ethical Issues in RLHF, even seemingly straightforward alignment goals such as the HHH principle are open to a variety of different interpretations, both within and across communities. With AI systems increasingly controlled by powerful actors with their own corporate and political agendas, this presents a steep challenge for ensuring systems serve broad populations of users and communities responsibly. Indeed, as Kalluri (2020) argues, whether AI systems are “safe” or “fair” misses the point, and the important questions are about how AI shifts power. While our focus has been on the HHH principle most used in current LLM RLHF-based fine-tuning, our work provides more general insight into issues of power.

Our analysis illustrates a more fundamental issue: value alignment is misconceived if seen and addressed through a mostly technical point of view. Values vary and are constantly renegotiated within societies and communities across time. Technology-first proposals for value alignment, such as RLHF, tend to neglect the role of democratic institutions in ethical deliberation through law and policy (Gansky & McDonald, 2022). Instead, it is well established that upholding values in technology design necessitates a broader lens that encompasses the design of the institutions and processes that structure the development and operation of technological systems (Koppenjan & Groenewegen, 2005) Institutional design refers to the design of structures that coordinate the positions, relations and behaviour of the parties that own and operate a system, but also those that are otherwise dependent on or affected by it. Process design refers to the need to design the processes that lead to the design of institutional arrangements or technological systems, including *designing the design process*. Process design is also vital in ensuring that procedural desiderata are met in processes, such as participatory, democratic and rule of law requirements (Nouws & Dobbe, 2024). As such, institutional, process and technology design are by no means separate efforts, nor does one strictly structure the others.

The core takeaway for those aiming to use RLHF methods to contribute to the upholding of key values in AI systems is that their efforts can both inform and be informed by efforts at the level of institutional and process design. Collectively, comprehensive design can aim to operationalise values, as well as to examine possible value conflicts resulting from misaligned interests.

### Embracing sociotechnical AI system safety and ethics

Within the comprehensive design lens, notions of safety and ethics can be instantiated through sociotechnical and systemic approaches. The need for system safety was recently acknowledged in the first International AI Safety Report (Bengio et al., 2025), following earlier responses to the narrow focus on technical interventions (Raji & Dobbe, 2020; Dobbe, 2022). A sociotechnical systems view of LLMs suggests that safety criteria and ethical assessments need to be situated, deliberated, and negotiated in the context of use, and span all layers of the sociotechnical system, including through organisational and institutional interventions (Nouws et al., 2023; Aler Tubella et al., 2023; Dobbe & Wolters, 2024). Recently, concepts and methods from system safety have re-emerged to address both safety concerns as well as broader ethical challenges in modern AI system development (Rismani et al., 2024).

Reframing AI safety as a sociotechnical discipline, we draw five core implications for feedback-guided methods and research. First, practically, RLHF interventions should adhere to the dynamic nature of safety problems. Recent advances in *reinforcement learning from hindsight simulation  (RLHS)* embrace this fact by providing evaluators with experience of the downstream outcomes of an interaction before being asked for feedback (Liang et al., 2025). Second, as the RLHS approach indicates, technical safety approaches need to engage with the sociotechnical nature of *system outcomes rather than outputs*. As such, technical AI Safety should grow towards a more mature engineering discipline that helps structure, build and secure system behaviours rather than model outputs. Third, AI safety should resist the framing of ‘frontier models’ as exhibiting inherently ‘dangerous capabilities’ (Anderljung et al., 2023). Normalising flawed models as ‘frontier﻿’ in policy promotes safety-washing and instils safety hazards in many more contexts (Dobbe, 2023), especially since the risks are imposed upon the public in large-scale experimentation. A sociotechnical AI safety discipline should motivate and prototype AI technologies that can be shown to have provably safe behaviours *within the sociotechnical context* of use and operation. Fourth, sociotechnical AI safety scholarship and practice should centre the need to curb the curse of flexibility (see the section on The Curse of Flexibility ). In order to eliminate or at least reduce the inherent safety limitations of overly complex software, we need to stop building or relying on overly large scale general-purpose models. Instead, the field should prioritise smaller, limited-purpose models and architectures that are more amenable to proper requirement engineering, to risk and ethical assessment, and which can cater to local needs and contexts, while requiring significantly fewer computational resources and thus having more limited ecological footprints (Rakova & Dobbe, 2023). Lastly, it is equally important to build safety-oriented scholarship that is open to the normative and political dimensions of safeguarding technological systems. Often, safety requirements are necessary but not clearly articulated, deliberated or negotiated with the proper actors. Operationalising any notion of safety for AI requires deliberation over the politics of development, as well as the context of deployment (Dobbe et al., 2021).

## Conclusion

In this paper, we challenge the claims made around the use of RLHF and the HHH principle (helpfulness, honesty, harmlessness) for achieving AI safety through alignment. Taking a sociotechnical perspective, we critique both the theoretical and practical elements of the approach, emphasising its limitations and inherent tensions.

While RLHF may be good for reinforcing anthropomorphic behaviour in LLMs, such fine-tuning techniques open up new problems.

Simple may indeed be memorable, but focusing on the HHH principle fails to encapsulate most of what is needed for building safe and ethical LLMs, and AI systems more generally. Beneath the thrust of RLHF techniques lies an oversimplification of the complexities of human diversity, behaviour, values, and ethics. A richer, more integrative perspective on safety and ethics is necessary, as outlined in the section Rebooting AI Safety and Ethics, in which technical design interventions are just one among the many needed efforts to build safer and ethically responsible AI systems .

## Data Availability

No datasets were generated or analysed during the current study.

## Footnotes

1 https://openai.com/blog/chatgpt

2 https://www.anthropic.com/index/claude-2

3 https://ai.meta.com/llama/
